# Tet Methylcytosine Dioxygenase 2 ( *TET2* ) Mutation Drives a Global Hypermethylation Signature in Patients With Pulmonary Arterial Hypertension (PAH): Correlation With Altered Gene Expression Relevant to a Common T Cell Phenotype

**DOI:** 10.1002/cph4.70011

**Published:** 2025-04-24

**Authors:** Charles C. T. Hindmarch, François Potus, Ruaa Al‐Qazazi, Benjamin P. Ott, William C. Nichols, Michael J. Rauh, Stephen L. Archer

**Affiliations:** ^1^ Department of Biomedical and Molecular Science (DBMS) Queen's University Kingston Ontario Canada; ^2^ Department of Medicine Queen's University Kingston Ontario Canada; ^3^ Queen's CardioPulmonary Unit, Translational Institute of Medicine (TIME), Department of Medicine Queen's University Kingston Ontario Canada; ^4^ Pulmonary Hypertension Research Group Center de Recherche de L'institut Universitaire de Cardiologie et de Pneumologie de Québec Quebec Canada; ^5^ Division of Human Genetics, Department of Pediatrics, Cincinnati Children's Hospital Medical Center University of Cincinnati College of Medicine Cincinnati Ohio USA; ^6^ Department of Pathology and Molecular Medicine Queen's University Kingston Ontario Canada

**Keywords:** DNA methylation, epigenetic, metabolism, reduced representation bisulfite sequencing (RRBS), T cell differentiation, transcription factor 7 (TCF7)

## Abstract

Epigenetic changes in gene expression due to DNA methylation regulate pulmonary vascular structure and function. Genetic or acquired alterations in DNA methylation/demethylation can promote the development of pulmonary arterial hypertension (PAH). Here, we performed epigenome‐wide mapping of DNA methylation in whole blood from 10 healthy people and 19 age/sex‐matched PAH patients from the PAH Biobank. Exome sequencing confirmed the absence of known mutations in PAH‐associated gene variants identifying subjects with or without mutations of TET2, a putative PAH gene encoding the demethylating enzyme, TET2. DNA of patients with PAH and no TET2 mutation was hypermethylated compared to healthy controls. Patients with PAH and a TET2 mutation had greater DNA CpG methylation than mutation‐free PAH patients. Unique Differentially Methylated Regions (DMR) were more common in patients with PAH with TET2 mutations (1164) than in PAH without mutations (262). We correlated methylome findings with a public PAH transcriptomic RNA dataset, prioritizing targets that are both hypermethylated in our cohort and downregulated at the RNA level. Relative to controls, functional analysis reveals enriched functions related to T cell differentiation in PAH patients with a TET2 mutation. We identified genes with downregulated expression that were hypermethylated in PAH patients (with or without a TET2 mutation). In both cases, a conserved T cell phenotype emerged. Pan‐chromosomal hypermethylation in PAH is greatest in patients with TET2 mutations. Observed hypermethylation of genes involved in the pathogenesis of PAH, such as EIF2AK4, and transcription factors that regulate T cell development, such as TCF7, merit further study and may contribute to the inflammation in PAH.

## Graphical abstract

1. Mutations in TET2 result in a 2. Pan‐chromosomal hypermethylation that results in 3. Abnormal expression of genes that participate in 4. Functions related to T‐cell differentiation and function, 5. Driving a T‐cell‐mediated increase in inflammatory cytokines, 6. That contributes to the development of PAH.

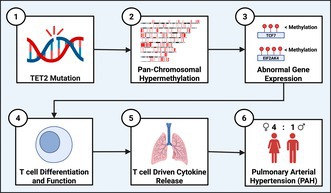

**Abbreviations**
BMPR2: bone morphogenetic protein receptor type 2
Chr: chromosome
CpG: 5'—C—phosphate—G—3
DMR: differentially methylated region
*EIF2AK4*
: eukaryotic translation initiation factor 2α kinase 4
FPAH: familial pulmonary arterial hypertension
GEO: gene expression omnibus
IPAH: idiopathic pulmonary arterial hypertension
PAH: pulmonary arterial hypertension
RRBS: reduced representation bisulfite sequencing
TCF7: transcription factor 7
TET2: tet methylcytosine dioxygenase 2
Th: T‐helper

## 1 Introduction

Pulmonary arterial hypertension (PAH) is a lethal vasculopathy characterized by obliterative remodeling, stiffening, and vasoconstriction in the pulmonary arterial circulation (Mocumbi et al. 2024). The etiology of PAH is heterogeneous and remains poorly understood, although it is characterized by increased inflammation (Al‐Qazazi et al. 2022; Potus et al. 2018) and immune dysfunction (Tomaszewski et al. 2021), which includes changes in T cell differentiation (Mansueto et al. 2021; Savai et al. 2012; Qiu et al. 2019), increased fibrosis (Al‐Qazazi et al. 2022; Tian et al. 2018, 2020), dysregulated angiogenesis (Potus et al. 2018; Ryan and Archer 2014), altered mitochondrial dynamics (favoring mitochondrial fission) (Dasgupta et al. 2020; Ryan et al. 2015; Sharp et al. 2015; Archer 2013; Marsboom et al. 2012), and mitochondrial metabolism (with a shift towards aerobic glycolysis or Warburg metabolism) (Tian et al. 2020; Ryan et al. 2015; Archer 2017; Rehman and Archer 2010).

At the genetic level, > 18 genes have been suggested to underlie heritable PAH (Lane et al. 2000; Rudarakanchana et al. 2002; Morrell et al. 2018; Southgate et al. 2020; Austin and Loyd 2014). These mutations are usually germline mutations that are inherited by an autosomal dominant mechanism with variable penetrance. Mutations are found in ~20% of patients with idiopathic PAH (IPAH) and 54% of patients with familial PAH (FPAH), leaving the etiology of most PAH cases unknown (Eyries et al. 2019). However, genetic mutations are not the only, or even the most common, means of altering gene expression. Epigenetic regulation represents a mechanism for molecular fine‐tuning of genome expression without changing DNA sequence. There are three major mechanisms of epigenetic regulation: DNA methylation, histone deacetylation, and the production of micro RNAs and long noncoding RNAs. Each of these mechanisms contributes to the pathogenesis of PAH (Kim et al. 2011).

Epigenetic regulation of gene expression often results from gene–environment interaction, and numerous such interactions predispose to PAH, including ingestion of amphetamines, anorexigens, exposure to high altitude, or infection with HIV (Almodovar et al. 2011; Montani et al. 2013). Although epigenetic signaling may be precipitated by gene–environment interactions, it is ultimately regulated by genes that control processes such as DNA methylation and demethylation. For example, changes in the expression and/or function of genes regulating DNA methylation, DNA methyltransferases (*DNMT*), and demethylation, Tet Methylcytosine Dioxygenase 2 (*TET2*), are crucial to determining the status of the methylome.

Epigenetic dysregulation of gene expression is implicated in the etiology of PAH (Tian et al. 2020; Gamen et al. 2016; Archer et al. 2010; Archer 2016; Chen, Dasgupta, et al. 2018; Hautefort et al. 2017; Kim et al. 2011). DNA methylation, mediated by DNMTs, results in the addition of methyl groups to CpG sites in gene promoter and enhancer regions. Hypermethylation of these regions often reduces gene transcription by interfering with the access of transcription factors. For example, superoxide dismutase (*SOD2*) (Mocumbi et al. 2024) and micro‐RNA‐126 (Potus et al. 2015) are regulated by a hypermethylated state in both human and preclinical models of PAH (Hautefort et al. 2017; Yan et al. 2020; Potus et al. 2015; Archer et al. 2010; Wang et al. 2018). Conversely, *TET2* catalyzes the removal of methyl groups (Moore et al. 2013; Quin et al. 2024). Recently, we demonstrated that predicted loss of function *TET2* mutations (somatic and germline) are likely a novel cause of PAH (Potus et al. 2020), a finding confirmed by others (Hiraide et al. 2022). However, the effects of *TET2* mutations on the methylome of PAH patients have not been explored.

In this study, we identify changes in global DNA methylation patterns in PAH patients with a mutation in *TET2* (Potus et al. 2020). We hypothesized that patients with a *TET2* mutation, which occurs in 0.39% of patients with PAH, would manifest a global hypermethylation relative to control subjects or patients with PAH that were not associated with a *TET2* mutation (Potus et al. 2020). We previously showed that *TET2* mutation disrupts gene expression patterns, contributing to a pathologic molecular environment that promotes hallmarks of PAH, notably increasing inflammation (reviewed in Mocumbi et al. (2024)).

To date, no comprehensive analysis of the effects of *TET2* mutation on DNA methylation in PAH has been performed. Here, using blood from our published cohorts of control subjects, patients with PAH and no mutation, and patients with PAH and a documented *TET2* mutation (Potus et al. 2020), we identify a global hypermethylation signature in patients with PAH. Furthermore, gene hypermethylation is exacerbated in patients with a *TET*2 mutation. We mined a publicly available transcriptomic database derived from patients with PAH and noted a correlation such that the genes we showed to be hypermethylated were downregulated in PAH. When we organized the differentially hypermethylated and downregulated genes according to gene function, we once again observe overarching functions related to T cell activity. Finally, comparison of the DMR profiles revealed DMRs that are directly related to *TET2* mutation versus those that are associated with PAH and independent of a *TET2* mutation. We note that the genes that were hypermethylated in PAH are predicted to pathologically regulate T cell function and differentiation.

## 2 Methodology

### 2.1 Ethics Statement

Blood samples were obtained from The National Biological Sample and Data Repository for PAH (PAH Biobank), maintained at Cincinnati Children's Hospital Medical Center (CCHMC). Patients are the same as were included in our original TET2 study (Potus et al. 2020). Control samples were obtained from consenting volunteers without PAH (TRAQ: 6014336, Queen's University). These studies were approved by the IRB of Queen's University, Canada (TRAQ: 6031198).

### 2.2 Patient Recruitment

**FIGURE 1:**
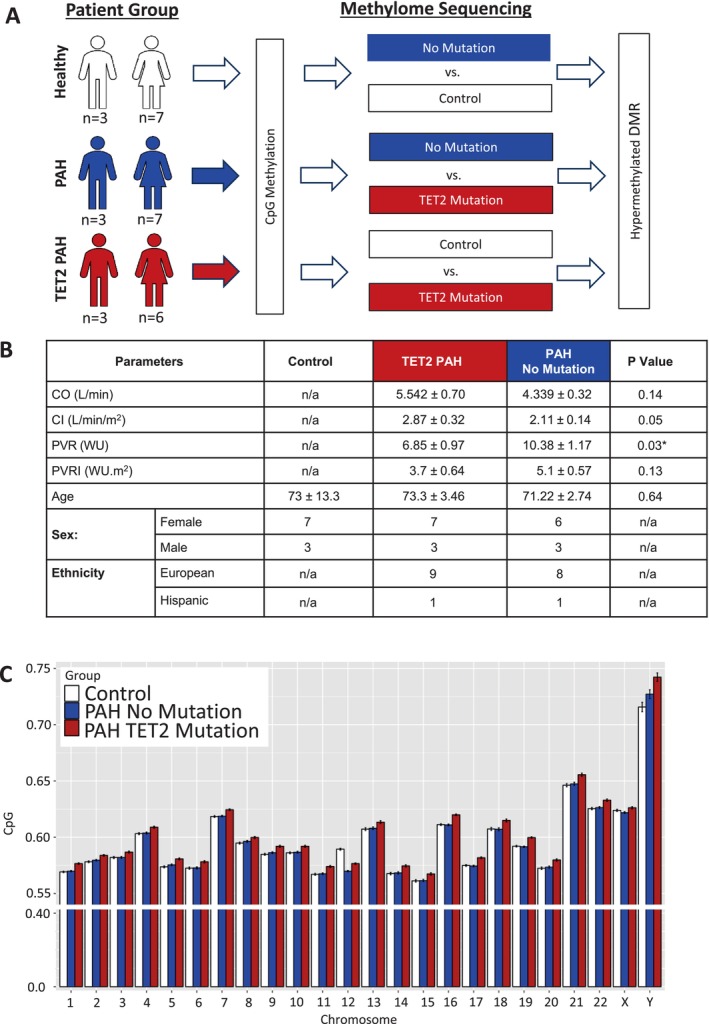
(A) Mutation of *TET2* is associated with panchromosomal hypermethylation. In total, blood from 19 patients with pulmonary arterial hypertension PAH were compared to blood from 10 age/sex‐matched controls. The PAH patients can be stratified into two distinct genotypes; 9 patients have a mutation in the Tet Methylcytosine Dioxygenase 2 (TET2) gene, and 10 have no known mutations. (B) Patients from which blood was taken are age/sex‐matched with controls. Values are expressed as: Mean (SD); BMI = body mass index, BSA = body surface area, CO = cardiac index, DPAP = diastolic pulmonary arterial pressure, IPAH = idiopathic pulmonary arterial hypertension, PAP = pulmonary arterial pressure, PCWP = pulmonary capillary wedge pressure, PVR = pulmonary vascular resistance, RAP = right atrial pressure, SPAP = systolic pulmonary arterial pressure. (C) CpG frequency without filter was overlaid on chromosomes for each of the three groups (Controls; PAH No mutation, PAH TET2 mutation), and presented as a histogram with the *y*‐axis reflecting MethDiff (the difference in average methylation ratios between the two groups at the specified site/region determined by subtracting the value for the second group, from the value for the first group).

None of the patients with PAH had mutations in known PAH genes, as determined by whole exome sequencing. Specifically, none of the 19 PAH patients had mutations in: *BMPR2*; *EIF2AK4*; *TBX4*; *ATP13A3*; *GDF2*; *SOX17*; *SMAD4*; *SMAD1*; *KLF2*; *BMPR1B*; *KCNA5*; *AQP1*; *ACVRL1*; *SMAD9*; *ENG*; *KCNK3*; *CAV1*. The PAH cohort includes 9 PAH patients carrying deleterious *TET2* mutations but having no mutation in any known PAH‐associated gene, and 10 PAH patients without mutation in *TET2* or other established PAH genes, as described (Potus et al. 2020). The healthy control group did not undergo sequencing. Cohorts were matched for disease severity and PAH subtype (60% IPAH and 40% associated PAH, APAH). The demographics of subjects in the whole methylome portion of this study appear in Figure 1.

### 2.3 Library Preparation and Data Analysis

Samples were processed and analyzed using RRBS (Zymo Research, Irvine, CA; Data S1). Independent Transcriptome data from the GSE38267 dataset (Chesné et al. 2014) was identified and extracted from the Gene Expression Database (GEO2R).

### 2.4 Data Availability

We have provided supplemental files that demonstrate the DMR filtering process.

### 2.5 Methyl‐MiniSeq Library Preparation

Genomic DNA was extracted from whole blood of 10 PAH patients with no *TET2* mutation, 9 patients with PAH and a *TET2* mutation, and 10 age, sex‐matched healthy controls. The healthy controls were not genotyped. Samples were processed and analyzed using Reduced Representation Bisulfite Sequencing (RRBS), a genome‐wide bisulfite sequencing technique (Zymo Research, Irvine, CA). Briefly, 250 ng of starting input genomic DNA was digested with 30 units of the restriction enzyme MSPL (NEB, Ipswich, MA) and then purified with Zymo Research DNA Clean & Concentrator‐5 (Cat#: D4003, Zymo Research, Irvine, CA). Fragments were ligated to pre‐annealed adapters containing 5'‐methyl‐cytosine instead of cytosine according to Illumina's specified guidelines (Illumina, California, USA). Adaptor‐ligated fragments ≥ 50 bp in size were recovered using the DNA Clean & Concentrator‐5 (Cat#: D4003, Zymo Research, Irvine, CA). The fragments were then bisulfite‐treated using the EZ DNA Methylation‐Lightning Kit (Cat#: D5030, Zymo Research, Irvine, CA). Preparative‐scale PCR was performed, and the resulting products were purified with DNA Clean & Concentrator‐5 (Cat#: D4003, Zymo Research, Irvine, CA) for sequencing on an Illumina HiSeq.

### 2.6 Sequence Alignments and Data Analysis

Primary analysis of methylation data was performed by Zymo (Zymo Research, Irvine, CA), using the following pipeline: Sequence reads from bisulfite‐treated Classic RRBS libraries were identified using standard Illumina base‐calling software and then raw FASTQ files were processed using TrimGalore 0.6.4 and FastQC 0.11.8 to assess the effect of trimming and overall quality distribution of the data. Alignment to the hg19 reference genome was performed using Bismark 0.19.0. Methylated and unmethylated read totals for each CpG site were called using MethylDackel 0.4.0. The methylation level of each sampled cytosine was estimated as the number of reads reporting a C, divided by the total number of reads reporting a C or T. Differentially methylated regions (DMR; minimum of 3 CpG sites; delta = 0.1; *p*‐threshold = 0.05 within a minimum of 50 bp) were identified for CpG islands with at least five read coverage and a 10% difference in methylation value between groups using a package called Dispersion Shrinkage for Sequencing data (DSS) (Park and Wu 2016).

### 2.7 Secondary Analysis

All secondary analysis was performed in R Studio (Vienna, Austria), unless otherwise indicated. CpG frequency (independent of statistical significance) was organized into chromosomes for each of the three groups (Controls; PAH No mutation, PAH *TET2* mutation) and presented as a histogram with the *y*‐axis reflecting MethDiff (the difference in average methylation ratios between the two groups at the specified site/region determined by subtracting the value for the second group, from the value for the first group). Differentially Methylated Regions (DMR) were organized (see above) and presented using KaryoploteR (Gel and Serra 2017), histograms generated using ggplot (Wickham 2016). Functional analysis of gene sets, performed using gProfiler (Raudvere et al. 2019) Upset plots were generated using UpsetR (Lex et al. 2014). Independent Transcriptome data from the GSE38267 dataset (Chesné et al. 2014) was identified and extracted from the Gene Expression Database (GEO; https://www.ncbi.nlm.nih.gov/geo/query/acc.cgi?acc=GSE38267).

### 2.8 Data Mining of a Published PAH Transcriptomics Study

We used the Gene Expression Omnibus (Edgar et al. 2002) to look for RNA sequencing datasets that we could use to correlate reduced gene expression in PAH with DNA hypermethylation in PAH, as identified in our DMR data. We searched using the phrase “pulmonary arterial hypertension” and we evaluated a published study by Chesné et al. A sub analysis was performed on microarray data from the blood of patients with chronic respiratory failure focused on whole blood samples from patients with PAH (*n* = 13; 8 female, 5 male; mean pulmonary arterial pressure 66.7 ± 17.17 mmHg) and healthy controls (*n* = 28; 19 female, 9 male; Chesné et al. 2014). GEO data was analyzed using GEO2R within the GEO portal, which relies on Linear Models for Microarray Analysis (limma) (Ritchie et al. 2015) to generate lists of differentially regulated genes from user submitted data. These data frames were then filtered for statistical significance (corrected *p* < 0.05) and to identify those genes that were downregulated relative to controls.

## 3 Results

For methylomic measurements, we used blood samples previously extracted from our published cohorts of patients with PAH and a *TET2* mutation (*n* = 9), or PAH without mutations of *TET2* (*n* = 10) versus healthy controls (*n* = 10) (Potus et al. 2020) (Figure 1A,B). Samples from patients with PAH were derived from the National Biological Sample and Data Repository for PAH (PAH Biobank). Females accounted for 70% of each group, and the mean ± SEM ages in the PAH, PAH with *TET2* mutation, and control groups were 71.6 ± 8.1, 69.1 ± 10.7, and 75.5 ± 6.7 years, respectively. Importantly, our published hemodynamic data from these patients showed that PAH associated with a *TET2* mutation is not hemodynamically more severe than idiopathic PAH; however, no subject with a *TET2* mutation was responsive to acute vasodilator challenge (vs. 13.2% responders in the balance of the PAH biobank cohort) (Potus et al. 2020).

We used Reduced Representation Bisulfite Sequencing (RRBS) to measure the methylation status of patients with PAH who have a *TET2* mutation compared to those who have PAH and no known mutation, or healthy controls. While we observed a 0.776% increase in methylation in PAH patients with no known PH gene mutation, relative to control subjects, there was a greater increase in methylation in the PAH *TET2* group (1.124%), compared to healthy controls (Figure 1C). At the chromosome level, significant hypermethylation in the PAH patients without any mutation was restricted to specific chromosomes (Chr 1, 2, 5, 7, 8, 9, 10, 12, 16, 22, and Y; Figure 1C). However, in patients with PAH and a *TET2* mutation, significant increases in methylation were observed on every chromosome (Figure 1C). A strength of this study is that all patients underwent whole exome sequencing as part of registration in the PAH biobank, and any patient with a mutation of a known PAH gene (except *TET2*) was excluded from analysis.

We clustered single CpG sites to define larger differentially methylated regions (DMRs) and thus identify target genes that would be predicted to have reduced expression, associated with excessive methylation. Once we identified DMRs from our data, we compared these lists of hypermethylated genes to a public transcriptomic dataset with a focus on genes with hypermethylated DMRs, identified from our data, and transcript downregulation in the independent dataset. All data and Venn comparisons are presented in Data S1 files which include a key.

### 3.1 Differentially Methylated Regions Between Control Versus PAH No Mutation

**FIGURE 2:**
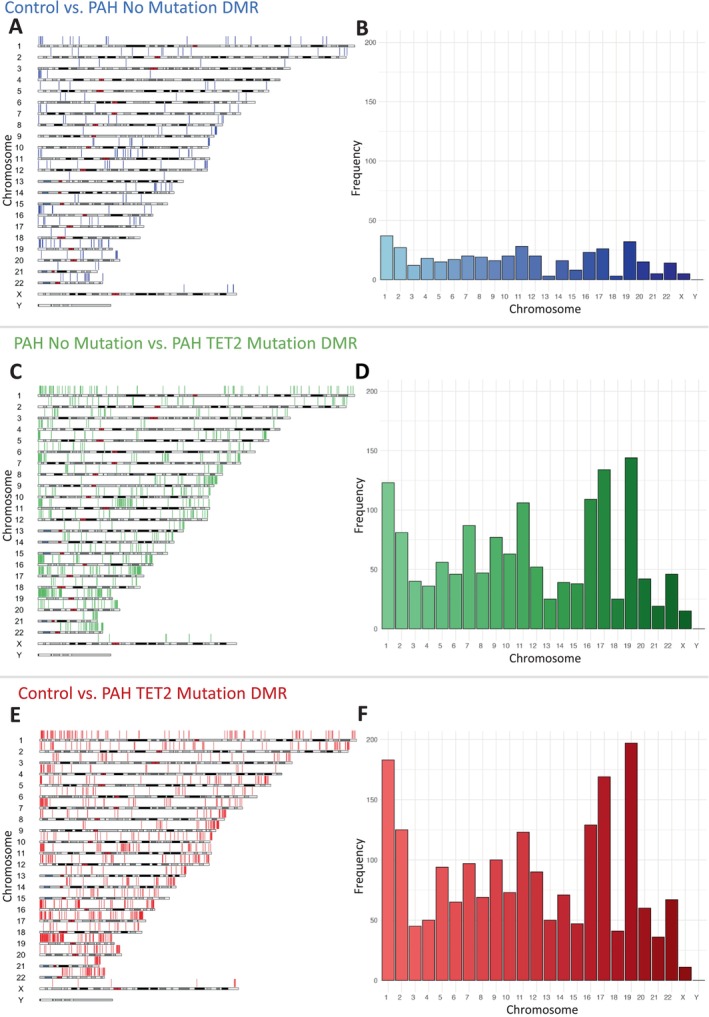
Hypermethylation of CpG groups in PAH is exacerbated by a *TET2* mutation. (A) DMR of patients with pulmonary arterial hypertension (PAH) (blue vertical lines) with no mutation compared to controls resolved by chromosome. (B) Frequency of DMRs from patients with PAH with no mutation compared to controls by chromosome. (C) DMR of patients with pulmonary arterial hypertension (PAH) compared to patients with PAH without mutations (green vertical lines) resolved by chromosome. (D) Frequency of DMRs of patients with pulmonary arterial hypertension (PAH) compared to patients with PAH without mutations by chromosome. (E) DMR of patients with PAH and a TET2 mutation compared to controls resolved by chromosome (red vertical lines). (F) Frequency of DMRs from patients with PAH with a TET2 mutation compared to controls by chromosome.

**FIGURE 3:**
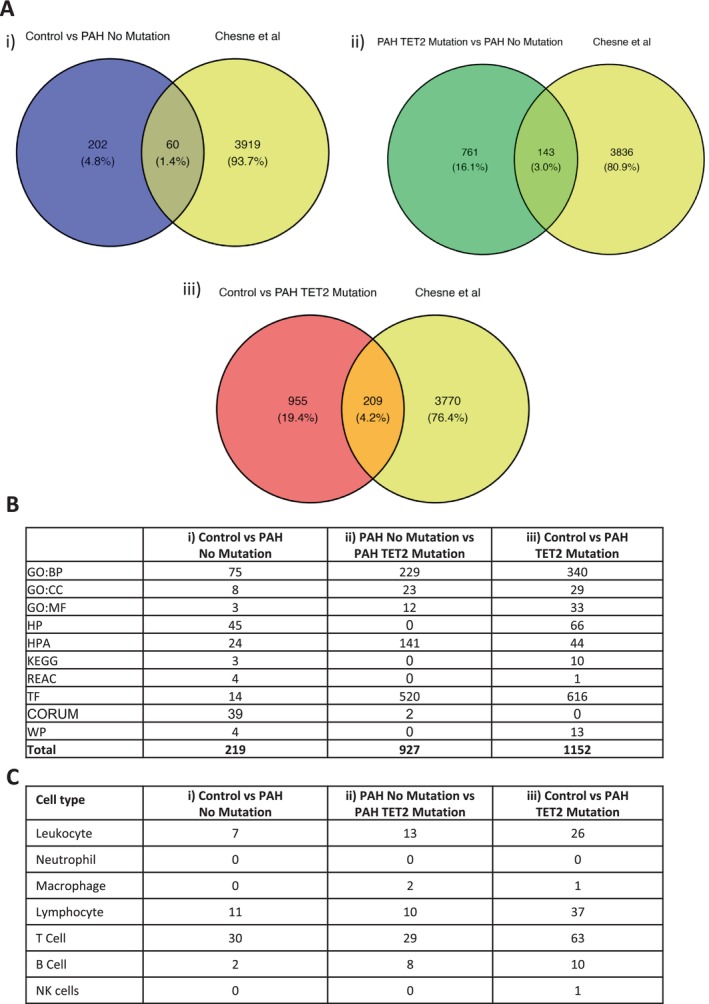
Regulation of molecular pathways in PAH subjects with or without *TET2* mutations A Comparison was made for each of our DMR lists ([Control vs. PAH TET2 mutation], [Control vs. PAH No mutation], and [PAH TET2 mutation vs. PAH No mutation]), and an independent transcriptome dataset extracted from the Gene Expression Omnibus (Edgar et al. 2002). Dataset GSE38267 (Chesné et al. 2014) was identified and extracted from the Gene Expression Database. This dataset focussed on whole blood samples from patients with PAH (*n* = 13; 8 female, 5 male; mean pulmonary arterial pressure 66.7 ± 17.17 mmHg) and healthy controls (*n* = 28; 19 female, 9 male). We used Venn diagrams to determine which of our DMR had genes that were downregulated in an independent cohort of PAH patients. Intersecting hypermethylated genes and downregulated genes from each comparison were passed through functional analysis, revealing large numbers of enriched terms for each DMR list. Filtering of these functions through the lens of cell type revealed a conserved pattern of T cell functions in our data, which is enriched in the blood of patients with PAH and a TET2 mutation. CORUM = the comprehensive resource of mammalian protein complexes, GO:BP = gene ontology (GO):biological process, GO:CC = GO:cellular component, HP = human phenotype, HPA = human protein atlas, KEGG = Kyoto Encyclopedia of Genes and Genomes, REAC = reactome pathway database, TF = transcription factors.

In patients with PAH but no known mutation, compared to controls, 675 DMRs were revealed, of which 262 were hypermethylated (Figure 2A and Data S1, S2). We wanted to compare genes with DMR identified in our data with a publicly available transcriptome dataset, with the aim of identifying common transcripts that are regulated in the blood of PAH patients. We re‐mined an existing, independent dataset created by Chesné et al. (GSE38267; Data S7) (Chesné et al. 2014), in which they performed transcriptomics in the blood of patients with PAH (*n* = 13) versus controls (*n* = 28) (Chesné et al. 2014) (Data S9–S17). We identified 60 common transcripts that are downregulated in the blood that also have hypermethylated DMRs according to our data (Figure 3Ai, Data S12). When we parsed this through functional analysis, we revealed 219 functions (Figure 3Bi, Data S13). Because these data are derived from blood, we searched for functions related to blood cells, including the terms ‘*leukocyte*’, ‘*lymphocyte*’, and specific cell types such as ‘*Neutrophil*’, ‘*macrophage*’, ‘*T cell*’, ‘*B cell*’, and ‘*NK cell*’ (Figure 3Bi). Most of these terms enriched around Lymphocyte (11 terms), and T cell (30 terms; Figure 3Ci).

### 3.2 Differentially Methylated Regions Between PAH TET2 Mutation and PAH With No Mutation

In PAH patients with a *TET2* mutation compared to PAH patients without mutation, 1917 DMRs were identified 904 of which were uniquely hypermethylated (Figure 2B; Data S3 and S4). Comparison to Chesné's published RNAseq data (Chesné et al. 2014) reveals 143 common genes (Data S15). Functional analysis of these genes reveals 927 enriched functions (Figure 3Aii, Data S16). We searched again for functions related to the term ‘*leukocyte*’, ‘*lymphocyte*’, and specific cell types such as ‘*Neutrophil*’, ‘*macrophage*’, ‘*T cell*’, ‘*B cell*’, and ‘*NK cell*’ (Figure 3Bii). Again, most of these terms enriched around Lymphocyte (11 terms), and T Cell (29 terms; Figure 3Cii).

### 3.3 Differentially Methylated Regions Between Control and PAH TET2 Mutation

In patients with PAH and a *TET2* mutation compared to controls, 2333 DMR were identified, including 1164 that were uniquely hypermethylated DMR (Figure 2C, Data S5 and S6). Comparison with Chesné's RNAseq data (Chesné et al. 2014) reveals 209 common genes (Figure 3Aiii, Data S9), which functionally fall into 1152 functions (Figure 3Biii, Data S10). When cell‐specific functions were filtered, there were 37 enriched lymphocyte terms and 63 enriched terms that related to T Cells (Figure 3Ciii).

### 3.4 DMRs Specific to *TET2* Mutation

**FIGURE 4:**
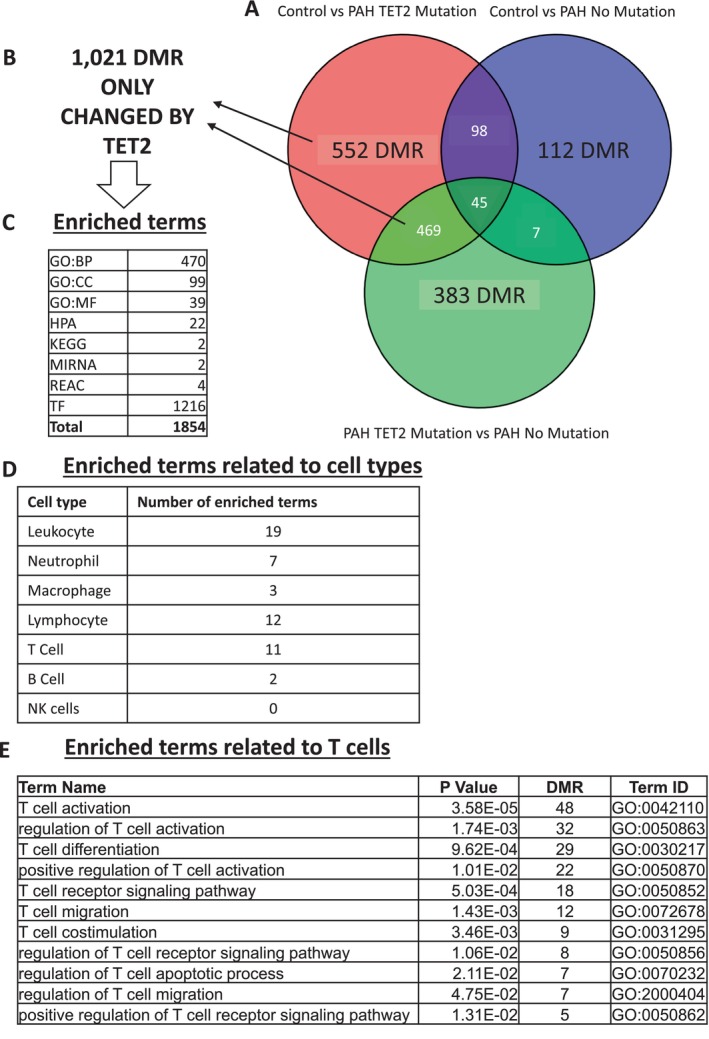
Pathway analysis of DMRs regulated by TET2 in peripheral blood reveals lymphocytes and T cells as enriched terms (A) Lists of Differentially Methylated Regions (DMR) ([Control vs. PAH TET2 mutation], [Control vs. PAH No mutation], and [PAH TET2 mutation vs. PAH No mutation]) were compared by Venn. (B) We identified 1021 DMR that were specific to TET2 mutation, (C) which resulted in the enrichment of 1854 functional terms. (D) When we filtered these terms to reveal those related to cell type, we found an enrichment of lymphocytes, specifically (E) T cells, of which 11 terms were enriched. CORUM = the comprehensive resource of mammalian protein complexes, GO:BP = gene ontology (GO):biological process, GO:CC = GO:cellular component, HP = human phenotype, HPA = human protein atlas, KEGG = Kyoto Encyclopedia of Genes and Genomes, REAC = reactome pathway database, TF = transcription factors.

**FIGURE 5:**
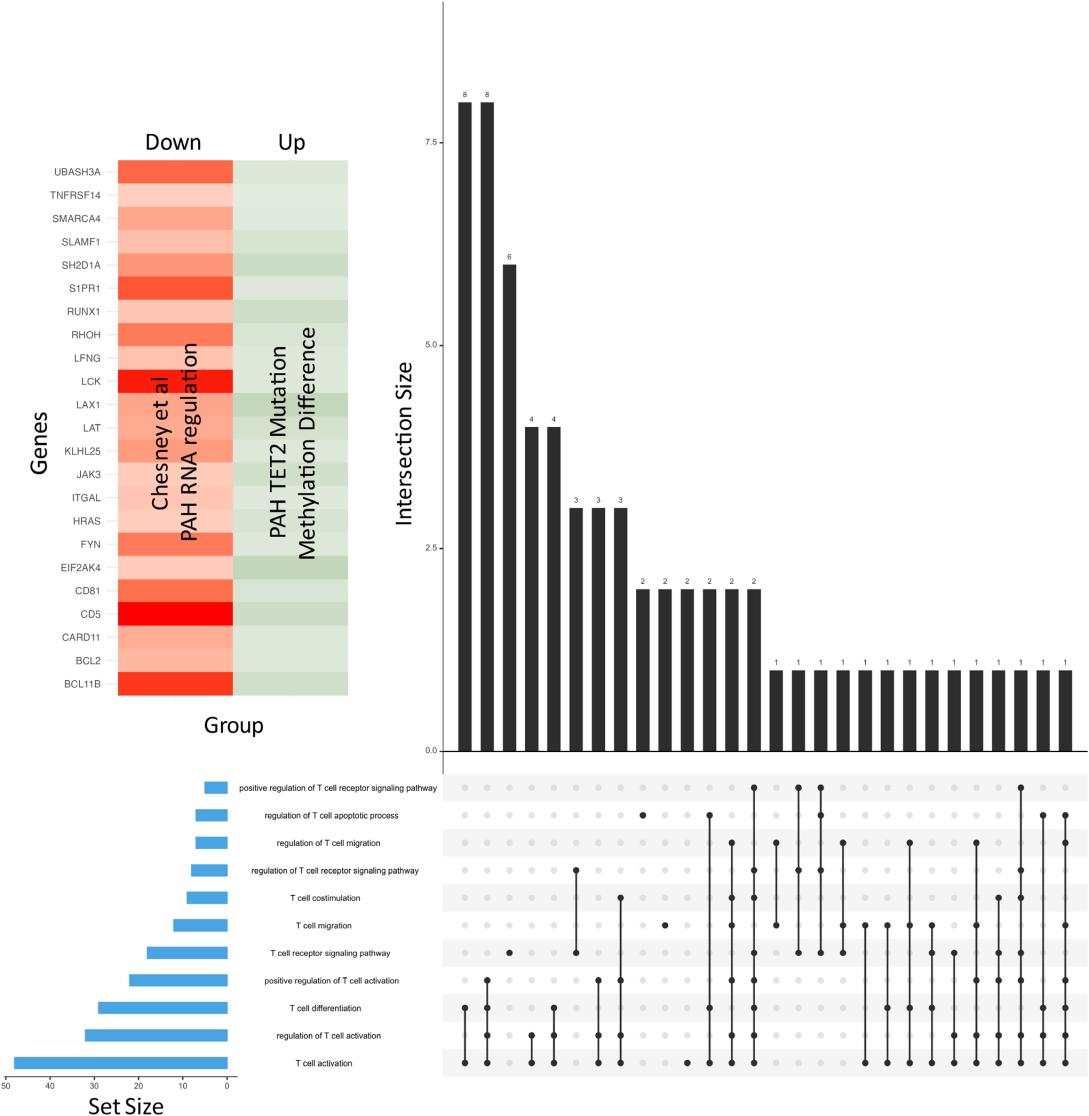
Examination of pathways comprised of hypermethylated and downregulated genes revealed effects on the expression of PAH‐relevant genes, including EIF2AK4 and the regulation of T cells. Upset plot reveals which genes are participating in the 11 overlapping T Cell functions that are specific to TET2 mutation (see Figure 4E). In total, 65 unique genes were identified as contributing to the 11 T cell functions, of which 23 DMRs are also regulated at the RNA level in an independent transcriptomic dataset (Chesné et al. 2014). The heatmap shows signal for RNA and methylation for each gene.

We compared the DMR from each subject group using a 3‐way Venn to identify those DMR that were uniquely the result of a mutation in the *TET2* gene (Figure 4A, Data S18–S26). From this comparison, we identified 1021 DMR that were under the control of *TET2* mutation (Figure 4B, Data S21). We wanted to know the functions that the *TET2*‐specific DMR participated in and identified 1854 enriched terms (Figure 4C, Data S22), which we organized into functions related to specific cell types (Figure 4D) and saw an enriched T cell phenotype of 11 terms (Figure 4E). These 11 enriched functions were enriched based on our identification of 65 unique DMR, 23 of which also have concordant gene expression profiles in Chesné's independent transcriptomic study (Chesné et al. 2014) (Figure 5).

### 3.5 Common DMRs Independent of *TET2* Status

**FIGURE 6:**
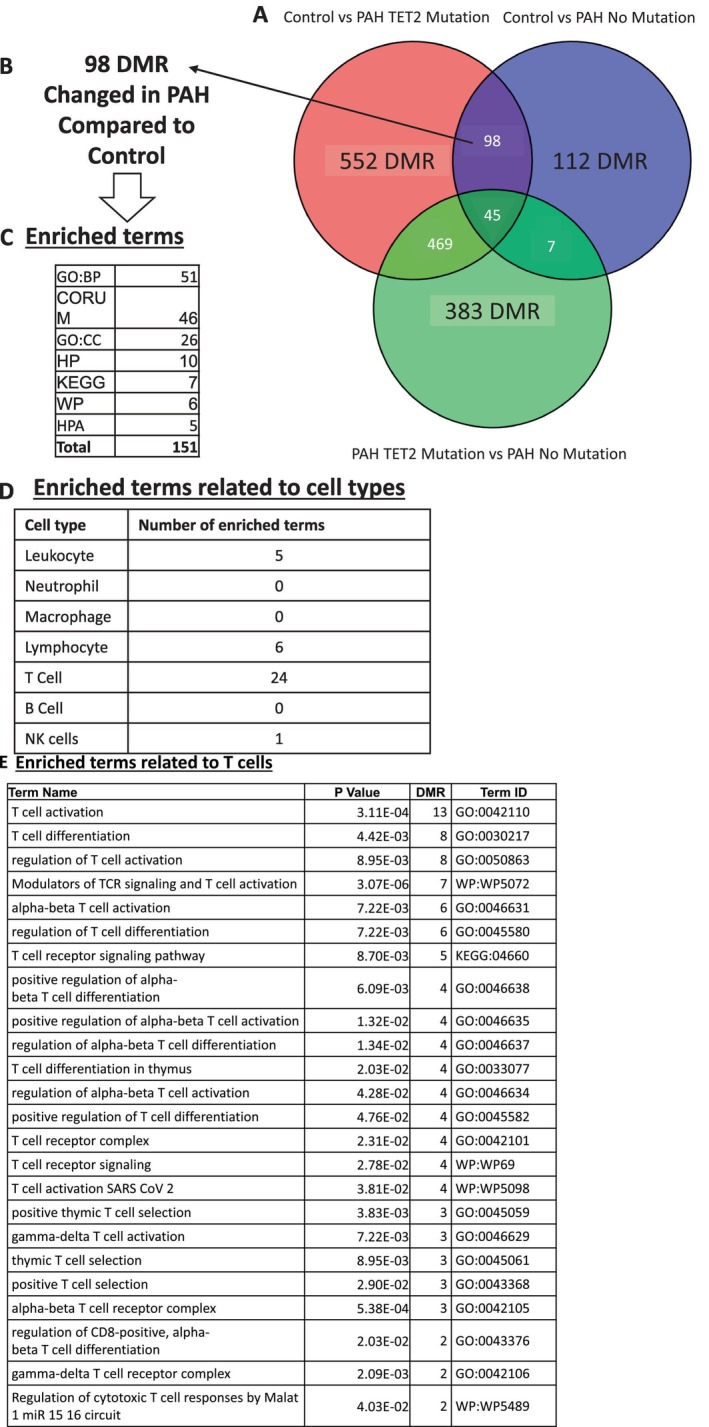
Pathway analysis of DMRs regulated by PAH in peripheral blood reveals enriched terms including lymphocytes and T cells (A) Lists of Differentially Methylated Regions (DMR) ([Control vs. PAH TET2 mutation], [Control vs. PAH No mutation], and [PAH TET2 mutation vs. PAH No mutation]) were compared by Venn. (B) We identified 98 DMR that were specific to PAH and independent of TET2 status, (C) which resulted in the enrichment of 151 functional terms. (D) When we filtered these terms to reveal those related to cell type, we found an enrichment of lymphocytes, specifically (E) T cells, of which 24 terms were enriched. CORUM = the comprehensive resource of mammalian protein complexes, GO:BP = gene ontology (GO):biological process, GO:CC = GO:cellular component, HP = human phenotype, HPA = human protein atlas, KEGG = Kyoto Encyclopedia of Genes and Genomes, REAC = reactome pathway database, TF = transcription factors.

**FIGURE 7:**
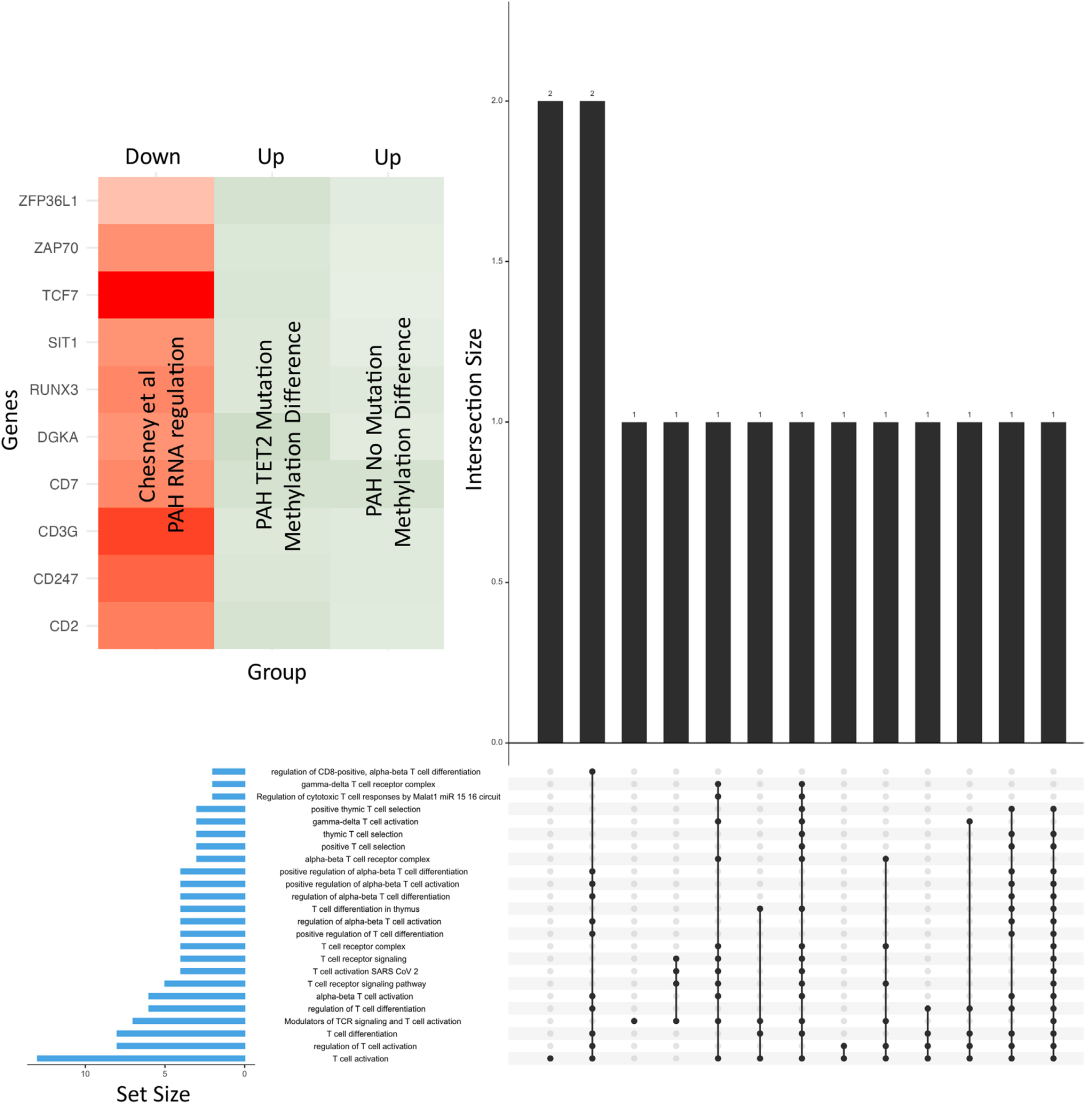
Pathway analysis of DMRs most regulated by PAH in peripheral blood reveals downregulation of the transcription factor TCF7 and enrichment of terms related to T cells. Upset plot to reveal which genes are participating in the 24 overlapping T Cell functions that are specific to PAH and independent of TET2 mutation status (see Figure 6E). In total, 15 unique genes were identified as contributing to the 24 T cell functions, of which 10 DMRs are also regulated at the RNA level in an independent transcriptomic dataset (Chesné et al. 2014). The heatmap shows signal for RNA and methylation for each gene in both comparisons. We highlight the gene TCF7 (transcription factor 7 which encodes TCF1 protein), a critical gene that regulates T cell differentiation *TCF7*.

We also wanted to understand which DMRs were enriched in PAH compared to controls, independent of *TET2* mutation status (Figure 6A). This analysis identified 98 common, PAH‐regulated targets (Data S24). When these genes were analyzed to determine the enriched functional terms, 151 were elucidated (Figure 6C, Data S25), of which 24 were related to T cell functions (Figure 6D,E), reflecting the contribution of 15 unique genes, of which 10 were concordantly down‐regulated in Chesné's independent transcriptomic dataset (Chesné et al. 2014) (Figure 7).

## 4 Discussion

Here, we performed epigenome‐wide mapping of DNA methylation in whole peripheral blood of 9 PAH patients with a mutation in the *TET2* gene, 10 age/sex matched PAH patients without any known PAH mutation, and 10 age/sex matched healthy controls, using RRBS. RRBS isolates GC‐enriched DNA fragments (the target for methylation) for sequencing (Emon et al. 2023). The advantage of RRBS is the broad genomic coverage achieved with modest requirements for total DNA versus conventional whole genome bisulfite sequencing. A weakness of this technique is that methylation in the large DNA segments which are GC‐poor is excluded.

The patient haemodynamic data demonstrates patients with a mutation in *TET2* have Group 1 PH, but their hemodynamics are not worse than subjects with idiopathic PH, as we have previously reported (Potus et al. 2020). We hypothesize that a mutation in the *TET2* gene deranges the same pathways that other genetic or environmental factors target to induce PAH; but does so through an epigenetically‐mediated mechanism. Our previously reported haemodynamic data for these patients confirm that PAH driven by a mutation in the *TET2* gene is no more severe than in those without the mutation; indeed, PVR was higher in those without the *TET2* mutation (Potus et al. 2020). While we are not proposing that these patients experience worse outcomes (Potus et al. 2020), their mechanisms(s) of developing PAH may be achieved through epigenetic modulation of conserved target genes and pathways across the genome that result in the same clinical outcome as seen in PAH patients who present without this gene mutation.

The major novel finding of this study is the existence of a pan‐chromosomal hypermethylation signature in PAH patients, which is more pronounced in those carrying *TET2* mutations, as would be predicted with a loss of TET2's demethylation function associated with the loss of function mutations previously noted in these patients (Potus et al. 2020). This study extends our prior work by confirming mutation of *TET2* does indeed alter the human methylome, beyond levels seen in PAH without *TET2* mutations. This observation is also consistent with the finding that mice with a hematopoietic deletion of *TET2* develop PAH spontaneously and attests to the adverse effects of DNA hypermethylation on the pulmonary vasculature (Potus et al. 2020).

Once we had identified methylated CpGs that expressed in control blood, versus blood from PAH patients with or without *TET2* mutation, we aggregated them into differentially methylated regions (DMR) and compared the following three groups: ([Control vs. PAH *TET2* mutation], [Control vs. PAH No mutation], and [PAH *TET2* mutation vs. PAH No mutation]). In line with our CpG data, we find that patients with PAH and a *TET2* mutation had the largest number of DMRs compared to control. Next, we performed an exploratory, correlative study to determine whether the genes that we demonstrated were hypermethylated in our cohort were also downregulated at the transcript level in an independent transcriptomic study cohort (Chesné et al. 2014). In this correlative exercise, where we compared methylome data to transcriptomic data derived from the blood of healthy controls and PAH patients (Chesné et al. 2014), the average age of controls and PAH cases was 42.5 ± 14.2 and 41 ± 15.3. By integrating our lists of DMR with this transcriptomic data, we identified genes that have concordantly hypermethylated regions (in our study) and reduced mRNA expression (Chesné et al. 2014). When the intersections of these comparisons were analyzed for functional enrichment of pathways related to blood cell types like leukocytes and lymphocytes, we revealed a conserved T cell phenotype across all PAH versus control comparisons, including in the PAH *TET2* mutation compared to controls.

There is an established role for T cells in PAH, which our methylation findings may partially explain. Naïve CD4^+^ T cells differentiate into T helper (Th) and T regulatory (Treg) cells, which have differential influences on the development of PAH. In PAH, there is an attenuation of anti‐inflammatory Treg cells, whereas numbers of inflammatory T‐helper (Th) cells are increased (reviewed in Rabinovitch et al. (2014)). Patients with IPAH have an increased number of T cells in their lungs (Mansueto et al. 2021; Savai et al. 2012). Within the population of Th cells, subtypes Th1 and Th17 cells induce an adverse, proinflammatory response in PAH that is mediated by a host of cytokines (Qiu et al. 2019). Furthermore, Th2 cells are increased in IPAH patients, and in a preclinical model of PAH in mice, suppression of Th2 cells attenuated the pathogenesis of PAH (Chen, Zuo, et al. 2018). Conversely, the abundance of Tregs is lower in both preclinical PAH models (Plecitá‐Hlavatá et al. 2023) and patients with PAH (Sada et al. 2016). It is interesting to note that in peripheral T cell lymphomas, mutations in *TET2* are common (Tigu and Bancos 2023) and *TET2* loss promotes CD8^+^ T cell differentiation (Carty et al. 2018). Thus, our observation that there is a significant increase in methylation, specifically in genes involved in T cell development, may have relevance to the pathogenesis of PAH. Taken together with the correlative finding of downregulation of gene expression in these same pathways in the study by Chesné et al. (2014), our data support the hypothesis that DNA hypermethylation contributes to the inflammatory phenotype of PAH.

We wanted to identify the DMR that were specifically under the control of *TET2*, so we looked for the intersection of DMRs between subjects with PAH *TET2* Mutation versus PAH No Mutation, and Control versus PAH *TET2* Mutation. This analysis identified 1021 DMR that are putatively under the control of TET2 or are responsive to the mutation of this gene. When this *TET2* DMR set was assessed using functional tools, we revealed 1854 enriched terms. When we interrogated this list of terms, we identified multiple enriched terms that relate to T cells, the result of regulation of 65 genes of which 23 are also regulated at the gene expression level.

One of the hypermethylated DMRs that is associated with mRNA downregulation involves eukaryotic translation initiation factor 2α kinase 4 (*EIF2AK4*), a known PAH gene, which underlies pulmonary veno‐occlusive disease (Austin and Loyd 2014; Best et al. 2014, 2017; Eyries et al. 2014). Single‐cell RNAseq analysis was performed on the lungs of a model of hereditary pulmonary veno‐occlusive disease (hPVOD; a rare form of PAH), created in rats using an *EIF2AK4* (alias: *GCN2*) knockdown. In this model, LAG3 T cells, proliferative T cells, and inflammatory cells (and genes) were all regulated by *EIF2AK4* knockdown (Bignard et al. 2023).

We are cognizant that some hypermethylated DMR may also participate in PAH pathology independent of *TET2* mutation, and so we looked at the 98 genes that are commonly hypermethylated between Control versus PAH *TET2* Mutation and Control versus PAH with no *TET2* Mutation. Passing these genes through functional analysis again revealed the conserved T cell phenotype. Interestingly, the 10 genes responsible for the 24 T cell pathways are all also downregulated in Chesné's RNA sequencing data from PAH patients. In contrast to the predicted adverse consequences of DMRs in patients with PAH and *TET2* mutations, none of the hypermethylated DMRs that were unique to non‐*TET2* PAH predicted pathologic changes in gene function.

Another identified hypermethylated genes, *TCF7* (transcription factor 7 which encodes TCF1 protein) is downregulated at the transcriptional level in the Chesné study (Chesné et al. 2014) and this would be predicted to exacerbate inflammation in PAH. TCF7 regulates T cell differentiation. *TCF7* is an end‐point mediator of the WNT signaling cascade, regulating gene expression in a β‐catenin dependent manner (Weber et al. 2011). *TCF7* simultaneously promotes T cell differentiation to Th2 or memory T cells, while suppressing proinflammatory Th1 and Th17 cells (Zhu et al. 2015). Therefore, if the expression of TCF7 is downregulated (epigenetically or otherwise), an increase in these proinflammatory T cells would be expected, consistent with the observed PAH phenotype, in which increased Th1 and Th17 cells are found (Chen, Dasgupta, et al. 2018). Therefore, our finding that *TCF7* is hypermethylated, and that the expression of *TCF7* is downregulated in the blood of PAH patients, implies that this may be a mechanism by which elevated Th1 and Th17 cells contribute towards the pro‐inflammatory phenotype common to this disease. While *TCF7's* expression is not necessarily dependent upon epigenetic modification induced by a *TET2* mutation, this remains a promising target because it is regulated and methylated in PAH irrespective of *TET2* status. We believe that methylation associated downregulation of TCF7 could be particularly relevant in PAH because the methylation of transcription factors has a greater impact on overall gene expression than does the methylation of other genes.

## 5 Limitations

This study has several limitations that are important to highlight since this is the first detailed analysis of the impact of a *TET2* mutations on methylation status. Firstly, the sample size was limited by PAH being an orphan disease and the low prevalence of *TET2* mutations in PAH (0.39%). Therefore, the study was insufficiently powered to address all questions of interest, such as whether there was a sexual dimorphism in gene methylation; however, no obvious sex differences among the control and PAH groups were noted.

Next, because we did not measure a transcriptomic signature from the same patients from whom we generated our methylome data, we had to rely on public datasets to make broad observations of the genes that are regulated in PAH and that may also be epigenetically regulated. The search for transcriptome blood data was not exhaustive, and in the data we mined, we do not have access to some important clinical information (such as comorbidities, risk factors, or genotypes). In the correlative exercise where we compared transcriptome data from the blood of healthy controls (HC) and PAH patients published by Chesné et al., the average age of HC and PAH cases was 42.5 ± 14.2 and 41 ± 15.3 years. While this is not age‐matched with our cohort, it does reduce the likelihood that these PAH patients are suffering from mutations to CHIP genes, including *TET2*, which are age‐related. Additionally, since the incidence of *TET2* mutations in PAH is < 1%, based on our prior publication (Potus et al. 2020), it is unlikely this external cohort would include a patient with a *TET2* mutation. We acknowledge that whereas increased gene methylation in patients with a *TET2* mutation in our own dataset reflects direct measurement, the potential impact of these methylated genes on gene expression is based on a correlation with mRNA expression in an independently published dataset. Thus, the imputed effects of gene methylation on T cell biology in PAH are correlative. To validate the potential effect of gene methylation on T cell regulation, validation studies in an independent pool of patients will be required. Lastly, while exonic and promoter methylation usually correlate inversely with gene expression (Li et al. 2018), hypermethylation can also increase transcription under some circumstances (Wan et al. 2015). A detailed analysis that stratifies DMR and gene expression RNA expression according to gene loci will be an important extension of these preliminary data.

## 6 Conclusions

Here, we present global methylation data captured using RRBS of blood from healthy controls compared to PAH patients, with or without a mutation in *TET2*. In patients with a mutation of *TET2* we found a pan‐chromosomal hypermethylation relative to control subjects and other PAH patients. When the CpGs were organized into differentially methylated regions, the genes that these DMRs converge on were associated with multiple functions, including T cell differentiation. Comparison of these hypermethylated genes with an independent RNAseq study of PAH patients versus controls resulted in an overlap in terms of both regulated genes and enriched functions. This study suggests: 1. that there is a causal link between *TET2* mutation and pan‐chromosomal gene hypermethylation; 2. there may be decreased expression of critical genes in PAH‐relevant GO pathways involving immunity and T cell differentiation, and that *TET2* mutation exacerbates this; 3. the epigenetic signal relevant to *TET2* mutation is identifiable in peripheral blood cells, suggesting it as a putative and clinically accessible biomarker; and 4. Hypermethylation of genes like TCF7 may contribute to differential T cell differentiation in PAH patients. More work is required to confirm which genes are dysregulated as a direct consequence of a mutation in *TET2* and to determine if these mutations occur primarily in the hematopoietic system, as in clonal hematopoiesis of indeterminate potential (CHIP) or are also found in the cells of the cardiopulmonary system.

## Conflicts of Interest

The authors declare no conflicts of interest.

## Supporting information


Data S1.

## Data Availability

The data that supports the findings of this study are available in the Supporting Information of this article.
